# Surgical Radioscopy under Red Light

**Published:** 1916-03

**Authors:** 


**Surgical Radioscopy Under Red Light.** J. Bergonie, Arch. d'Elect. Med., Dec. Operations requiring localization by X-rays require either that the surgeon shall work in the dark or be guided by an observer with a fluoroscope who cannot see the field of operation, or that the work shall proceed from recollections of previous X-ray examinations, or shall be subject to the delay of aljustment of the eyes to light and dark, for alternate surgical manoeuvres and fluoroscopy. For three months, operations requiring X-ray guidance have been performed in a room lighted with 20 25-candle power bulbs in the ceiling, above a double thickness of commercial ruby red glass. The light is sufficient, there being no shadows and on account of the contrast between the red rays and the green and yellow rays of the X-ray tube, fluoroscopy, by means of a screen protected by sterile tissue, can be resorted to without delay.

